# Editorial: mHealth Interventions for Improving Adolescent Sexual and Reproductive Health

**DOI:** 10.3389/frph.2022.945455

**Published:** 2022-07-04

**Authors:** Amy K. Johnson, Ososese Enaholo, Lynda Stranix-Chibanda, Stephanie R. Partridge

**Affiliations:** ^1^Feinberg School of Medicine, Northwestern University, Evanston, IL, United States; ^2^Division of Adolescent Medicine, Department of Pediatrics, Ann & Robert H. Lurie Children's Hospital of Chicago, Chicago, IL, United States; ^3^Faculty of Medicine and Health Sciences, University of Zimbabwe, Harare, Zimbabwe; ^4^Faculty of Medicine and Health, The University of Sydney, Darlington, NSW, Australia

**Keywords:** mobile health (mHealth), adolescent health, sexual health, reproductive health, sub-Saharan Africa

Gaps in adolescent sexual and reproductive health have led to the development, implementation, and evaluation of alternative methods to impact health outcomes for young people. With the current proliferation of technology, researchers see the use of mobile health (mHealth) as a vital tool in reaching adolescents and facilitating behavior change among this population. mHealth can be described as a subcategory of electronic health (eHealth), specifically delivered on mobile and wireless technologies to support interventions (1). The use of mHealth has shown to be promising in addressing several barriers that impact the sexual and reproductive health of young people. Amid the COVID-19 pandemic, the limitations set on social interactions and limited access to traditional, in-person health services stand as a major barrier for adolescents to promote their health. With an increase in smartphone usage globally, mHealth can benefit young people as it capitalizes on a familiar medium and mode of communication. mHealth *via* mobile phones is a particularly salient approach in sub-Saharan Africa, as mobile phone ownership among adolescents is >77% and internet penetration is high in many countries (2). This Editorial introduces three manuscripts published as a collection in response to the Research Topic: *mHealth Interventions for Improving Adolescent Sexual and Reproductive Health*. These projects, all based in sub-Saharan Africa, present different approaches to using mHealth as a driver of positive change for the sexual and reproductive health for young people, with an emphasis on adolescent populations affected by HIV.

- Summary of *Use of mHealth Solutions for Improving Access to Adolescents' Sexual and Reproductive Health Services in Resource-Limited Settings: Lessons From Zimbabwe* (Dhakwa et al.)

Dhakwa et al. assessed the effectiveness of mHealth in improving the linkage to reproductive health and HIV services as well as the turnaround time for referral completions among adolescent girls and young women (AGYW) in Zimbabwe. AGYW were enrolled through the Determined, Resilient, Empowered, AIDS-free, Mentored, and Safe (DREAMS) program and had automatic reminders sent to their phones to facilitate access to services through text messages and a paper-based system. The study observed 8,800 AGYW aged 10–19 years, with 4,355 referred through mHealth and 4,445 through the paper-based system. Dhakwa et al. found that over 95% of AGYW referred through mHealth completed referrals, compared to 87.8% that were referred through the paper-based system. The median for referral completion time was 1 day for mHealth and 11 days for the paper-based system. AGYW referred by mHealth were almost 18 times more likely to complete their referral compared to those referred by the paper-based system. The study concluded that the utilization of mHealth is beneficial in increasing the uptake of reproductive health and HIV services and reducing the referral completion time for AGYW in Zimbabwe.

- Summary of *PEERNaija: A Gamified mHealth Behavioral Intervention to Improve Adherence to Antiretroviral Treatment Among Adolescents and Young Adults in Nigeria* (Ahonkhai et al.)

In Nigeria, the use of mHealth was examined as an intervention to improve Antiretroviral Treatment (ART) adherence for adolescents and young adults living with HIV (AYALWH). Ahonkhai et al. used the Integrate, Design, Assess and Share (IDEAS) framework to develop the mHealth application, PeerNaija. As part of the IDEAS framework, a comprehensive development process was conducted which included targeting specific behavior *via* behavioral theory, prototyping, and direct engagement and feedback from AYALWH. To address common barriers to ART adherence such as forgetting to take ART, poor executive functioning, poor social support, and the indirect cost of clinic-based interventions, the PeerNaija application highlighted the social and peer-based nature of its features. Key features include reminders to take medication, ability to record doses taken, a chat-based social feature, and several gamification features and mechanics, including points, progress feedback, leaderboards, badges, and avatars. The gamification features further encourage adolescents to engage with the app while positively changing their behaviors through setting goals, building the capacity to overcome challenges, giving feedback, and comparing their progress. PeerNaija also stands as a mobile personal health record application that shares data with the OpenMRS electronic health record application used in Nigeria. Though PeerNaija is currently undergoing formal implementation and evaluation, theory based mHealth applications that utilize social incentives have the potential of improving adherence to ART for AYALWH in Nigeria.

- Summary of *Khuluma: Using Participatory, Peer-Led and Digital Methods to Deliver Psychosocial Support to Young People Living With HIV in South Africa* (Atujuna et al.)

Much success has been seen in the utilization of mHealth in South Africa, as it stands as a driver in the increase of universal health coverage. Atujuna et al. sought to evaluate the usage of mHealth as a tool to improve the life outcomes for adolescents living with HIV (ALWH) in South Africa. Through the construction of Khuluma, a psychosocial and peer-to-peer mHealth intervention, text messaging was used to facilitate support groups for ALWH. This study enrolled 52 ALWH aged 15–20 years old, to participate in a 6-month pilot study. The participants were given new phones that had text message functionality and a registered SIM card. Participants were asked to decide on personal pseudonyms and each was given a participant ID number that was used to identify the phone. Khuluma utilized a combined approach for the facilitation of the virtual support groups, which included professional counselors and peer mentors. After an assessment of the group conversations, the themes identified were social support, stigma and disclosure, and medication adherence. Khuluma received high levels of engagement with 100% of participants sending at least one message. Atujuna et al. found the success of this intervention to be attributed to the feasibility of delivery of social support *via* text, adolescents feeling of ownership over the technological space, facilitation by both professional counselors and peer mentors, and the range of conversation topics that do not pertain to HIV. The study suggests that mobile phone-based support interventions are useful in providing safe spaces for ALWH to express themselves and provide support for each other.

mHealth has been shown to be an effective method to engage adolescents in interventions that support behavior change. In all three studies, the feasibility of using mobile platforms with adolescents to share health information and collect data is well-documented. In *Khuluma: Using Participatory, Peer-Led and Digital Methods to Deliver Psychosocial Support to Young People Living With HIV in South Africa* of Atujuna et al. and *Use of mHealth Solutions for Improving Access to Adolescents' Sexual and Reproductive Health Services in Resource-Limited Settings: Lessons From Zimbabwe* of Dhakwa et al. adolescents expressed high levels of acceptability and satisfaction with mHealth. Pertinent to establishing acceptability and ensuring utility among adolescents, is using a participatory approach in formative stages of mHealth design. Ahonkhai et al., Atujuna et al., and Dhakwa et al. used co-design principles and participatory methods to reflect adolescents' desired mHealth attributes, furthermore these studies overlapping timelines represent an opportunity to truly tailor mHealth to intended audiences. All three studies identified access to mobile data as a possible limitation for adolescents. Prior research indicates that we should not assume adolescents have the requisite literacy, stable internet connectivity, and access to power upon which this technology depends (3). Further, as mHealth interventions become increasingly available among adolescents in sub-Saharan Africa, attention to equity should be the top priority (3). Despite some challenges, all authors unanimously recommended the use of mHealth in educating and improving the sexual and reproductive health of adolescents.

## Author Contributions

All authors provided critical feedback and helped shape the editorial and have reviewed, revised, and confirmed the final version of the editorial and have contributed equally.

## Conflict of Interest

The authors declare that the research was conducted in the absence of any commercial or financial relationships that could be construed as a potential conflict of interest.

## Publisher's Note

All claims expressed in this article are solely those of the authors and do not necessarily represent those of their affiliated organizations, or those of the publisher, the editors and the reviewers. Any product that may be evaluated in this article, or claim that may be made by its manufacturer, is not guaranteed or endorsed by the publisher.
